# In Utero Exposure to Particulate Air Pollution during Pregnancy: Impact on Birth Weight and Health through the Life Course

**DOI:** 10.3390/ijerph17238948

**Published:** 2020-12-01

**Authors:** Susanne Steinle, Helinor J. Johnston, Miranda Loh, William Mueller, Sotiris Vardoulakis, Kraichat Tantrakarnapa, John W. Cherrie

**Affiliations:** 1 Institute of Occupational Medicine, Research Avenue North, Edinburgh EH14 4AP, UK; susanne.steinle@iom-world.org (S.S.); miranda.loh@iom-world.org (M.L.); will.mueller@iom-world.org (W.M.); Sotiris.Vardoulakis@anu.edu.au (S.V.); 2 Institute of Biological Chemistry, Biophysics and Bioengineering, School of Engineering and Physical Sciences, Heriot Watt University, Riccarton, Edinburgh EH14 4AS, UK; H.Johnston@hw.ac.uk; 3 National Centre for Epidemiology and Population Health, Research School of Population Health, Australian National University, Canberra ACT 2601, Australia; 4 Department of Social and Environmental Medicine, Faculty of Tropical Medicine, MAHIDOL University, 420/6 Ratchawithi Road, Ratchathewi, Bangkok 10400, Thailand; kraichat.tan@mahidol.ac.th

**Keywords:** birth weight, LBW, health risk, air pollution, particulate matter, NCD

## Abstract

In high-income countries, and increasingly in lower- and middle-income countries, chronic non-communicable diseases (NCDs) have become the primary health burden. It is possible that in utero exposure to environmental pollutants such as particulate matter (PM) may have an impact on health later in life, including the development of NCDs. Due to a lack of data on foetal growth, birth weight is often used in epidemiologic studies as a proxy to assess impacts on foetal development and adverse birth outcomes since it is commonly recorded at birth. There are no research studies with humans that directly link PM exposure in utero to birth weight (BW) and subsequently, the effects of lower BW on health outcomes in old age. It is, however, plausible that such associations exist, and it is thus important to assess the potential public health impacts of PM across the life course, and it is plausible to use birth weight as an indicator of risk. We therefore split this narrative review into two parts. In the first part, we evaluated the strength of the evidence on the impact of PM exposure during the entire pregnancy on birth weight outcomes in ten meta-analyses. In the second part, we reviewed the literature linking lower birth weight to childhood and adult chronic cardiovascular disease to explore the potential implications of PM exposure in utero on health later in life. Within the reviewed meta-studies on birth weight, there is sufficient evidence that PM pollution is associated with lower birth weight, i.e., the majority of meta-studies found statistically significant reductions in birth weight. From the second part of the review, it is evident that there is good evidence of associations between lower birth weight and subsequent cardiovascular disease risk. It is thus plausible that in utero exposure to PM is associated with lower birth weight and persisting biological changes that could be associated with adverse health effects in adulthood. Based on the reviewed evidence, however, the magnitude of later life cardiovascular health impacts from in utero exposure and its impact on BW are likely to be small compared to health effects from exposure to particulate air pollution over a whole lifetime.

## 1. Introduction

David Barker pioneered the foetal origins of adult disease hypothesis [1], and his research identified low birth weight (LBW) (<2500 g) as a potential indicator of adult chronic disease [2]. Stresses during pregnancy may induce physiological changes that can manifest later in life [3]. For example, since the 1960s, it has been known that cigarette smoking during pregnancy is associated with spontaneous abortion, stillbirth, and neonatal death, and more recently, evidence has emerged of possible risks of congenital anomalies, childhood neurobehavioural disorders, and other complications [4]. Smoking during pregnancy and maternal exposure to second-hand cigarette smoke also increases the risk of preterm birth (PTB), LBW and/or term low birth weight (TLBW) [5,6].

Like second-hand smoke, in utero exposure to particulate matter (PM) air pollution may influence the health of the foetus. There is evidence that outdoor PM exposure is associated with LBW, PTB and change in birth weight (BW) [7,8]. In addition, an association has been established between adverse birth outcomes and PM emissions from household combustion of solid fuels [9], as well as air pollution from agricultural burning and wildfires [10].

Rodent studies have shown that PM can cause reproductive and developmental toxicity following pulmonary exposure; reviewed by Chaudhuri et al. [11] and Ema et al. [12]. In vivo research has shown the adverse effects of particles on various parameters following the pulmonary exposure of pregnant mice, including but not limited to; sexual development, fertility (e.g., sperm production and activity, testicular weight, hormone levels such as testosterone), litter parameters (e.g., number of offspring, foetus weight, gender ratio, gestation length), and abnormal deliveries (e.g., abortion) [11]. The exact mechanism(s) underlying PM toxicity to the developing embryo/foetus have not been identified, although it has been suggested that the toxicity may be driven by direct and indirect effects [12,13,14]. Direct effects are associated with the translocation of particles or components associated with PM (e.g., PAHs, metals) across the pulmonary barrier to the blood circulation and then the placenta, allowing them to reach the foetus where their ability to stimulate inflammatory and oxidative responses drives their adverse effects. Indirect effects may arise from translocated particles causing placental dysfunction or the activation of inflammatory and oxidative responses by PM in the lung leading to circulating inflammatory mediators, which affect the placenta or the foetus [12,13,14,15].

There have been a number of research studies that have identified associations between in utero exposure to PM and adverse health; for example, reduction in lung function at age 8 [16] increased the risk of lower respiratory tract infection at age 1 [17], brain structural changes in the cerebral cortex [18], increased systolic and diastolic blood pressure age 4–6 [19], and increases in carotid artery arterial stiffness in young adults with maternal PM_2.5_ exposure [20]. However, to date, there are no human research studies that directly link PM exposure in utero, its effect on BW and subsequent impacts of BW on later adult life adverse health outcomes. It is, however, plausible that such associations exist, and it is therefore important to assess the potential public health impacts across the life course associated with in utero exposure to PM and its effects on BW. If such associations exist, this would imply an even larger societal impact from PM exposure than over the 100 million disability adjusted life years (DALYs) as currently assessed by the Global Burden of Disease [21], especially for middle-income countries such as India, China and Thailand, where PM levels are high. We focus on BW as an effect of in utero exposure and as a factor for later life health effects, because it is recorded at many births worldwide and is often used as a proxy for in utero development and healthy birth outcomes. The availability of BW data makes it an important indicator for estimating health impacts of in utero exposure on later life health.

There is evidence from Barker’s original research and subsequent studies that there is an association between low birth weight and cardiovascular disease (CVD) [1]. Several authors have speculated on the biological mechanisms behind the association, including inflammation and oxidative stress causing foetal programming of CVD [22,23]. The aim of this review was to evaluate the human epidemiological evidence for an association between exposure to particulate air pollution in utero and its effects on BW. In a next step, we assess the possible cardiovascular impacts in old age, where there is stronger evidence of an association with BW, on health due to low/lower BW to decide whether it is important to include such exposures in health impact assessment studies of outdoor air pollution.

## 2. Methods

**Figure 1:**
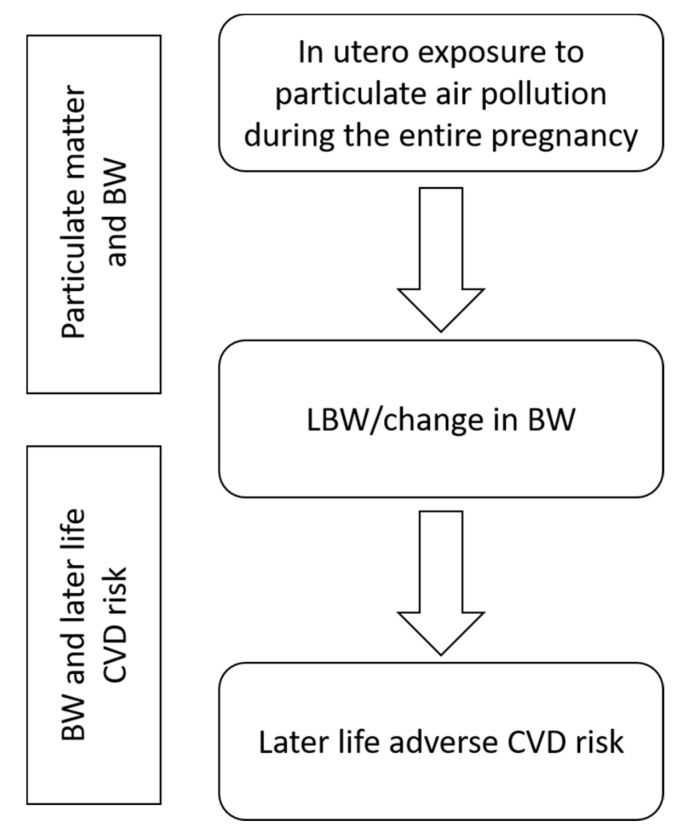
Schematic display of the structure of this review. In a first step, the impact of in utero particulate matter (PM) exposure on birth weight (BW) is assessed, and in a second step, the impact of low or lower birth weight (LBW) on later life cardiovascular disease (CVD) risk.

This review is structured in two parts, as shown in Figure 1. We searched Web of Science and PubMed for meta-analyses of particulate air pollution (PM_2.5_—particulate matter with aerodynamic diameter less than 2.5 μm, and PM_10_—particulate matter with aerodynamic diameter less than 10 μm) and LBW or BW. Using the following search string: “meta analysis” AND (“PM” OR “PM2.5” OR “PM10” OR “particulate matter”) AND (birthweight OR “birth weight” OR “Low birth weight”). Searches were restricted to the years 2008–2020 and English language only.

We extracted the meta-effect estimates from each meta-analysis along with the number of studies and potential confounders included in the various subgroup meta-analyses, as well as information about the input studies (we refer to the individual, original studies that were used in the meta-analyses as input studies throughout the text), such as study country, population size and exposure levels. We chose to focus on exposure estimates across the entire pregnancy, since all meta-analyses investigated this period for the outcomes of interest. Meta-effect estimates from all studies were standardised to represent risks for a 10 µg/m^3^ increase in PM exposure, where possible. Estimates were excluded from studies where the value of the risk increment (e.g., IQR) was not reported [24].

We also report the heterogeneity (I^2^), provided by the meta-analyses alongside their risk estimates to give an indication of the inconsistency of findings across the contributing studies. The I^2^ value ranged from 0% to 100% and provided a measure of the degree to which the confidence intervals of the contributing studies overlapped [25]; further, the I^2^ indicates what proportion of the observed variance would remain if sampling error could be eliminated.

We carried out similar searches of the literature for meta-analyses of LBW or BW and later-life health impacts. A preliminary search identified a recent umbrella review of previous systematic reviews and meta-analyses of birth outcomes and subsequent health status [26]. In this review, thirty-nine papers provided data for 78 associations between BW or small for gestational age and various health outcomes. These studies identified convincing or highly suggestive evidence of associations between LBW and the increased risk of perinatal mortality in low and middle-income countries, wheezing illness in childhood, coronary heart disease and all-cause mortality, although they considered the latter finding was most likely due to the observed association with CVD mortality [26]. There was also highly suggestive evidence for a number of other health conditions, but we have chosen to focus on BW and CVD for this review because of the relatively strong epidemiological evidence for this association. We searched Web of Science and PubMed for meta-analyses of (search string: “meta analysis” AND ((birthweight OR “birth weight” OR “low birth weight”) AND (“heart disease” OR “cardiovascular disease”)). Searches were restricted to the years 2008–2020 and English language.

## 3. Results

### 3.1. Particulate Matter and Birth Weight

We identified ten relevant meta-analyses, of which five investigated the impact of PM exposure in utero during the entire pregnancy (EP) on LBW only, one investigated changes in BW only, and the remaining four investigated both. Of the nine studies looking at LBW, only three [24,27,28] included the assessment of TLBW. Nine meta-studies were based on literature reviews and an analysis of data provided in the identified studies. The other study [27] worked directly with a group of partner-centres who together developed a common analytical protocol for re-analysing already existing datasets; this meta-analysis combined published studies and unpublished data.

All meta-studies reviewed applied different criteria for selecting their input studies, for example, some excluded study designs such as time-series, case-reports, case-series [29,30], others reviews and repeat literature [31,32]. Two meta-studies restricted the inclusion to cohort and case-control design [33] or cohort design only [34]. Three studies applied no restrictions [7,24,28]. The number of individual studies included in the various meta-analyses ranges between 2 and 29 studies. The reviewed meta-analyses reported meta-odds ratios (ORs), except for Li et al. [32], who reported relative risks (RR) of PM exposure and BW (as either a binary or continuous outcome) with 95% confidence intervals (CI). With LBW or lower BW being relatively rare, the risk measures (ORs and RRs) are approximately comparable. Heterogeneity ranged between 0.0% and 99.0% in all meta-analyses.

Nine of the studies assessed the impact of in utero PM_2.5_ exposure on LBW (Table 1); similar results were found in the studies using PM_10_ as their exposure metric and these are summarised in Supplementary Materials Table S1. Significant increases in ORs/RRs (95% CI) were reported by Zhu et al. [31] (1.05 [1.02, 1.07]), Li et al. [32] (1.08 [1.04, 1.12]), Sun et al. [7] (1.09 [1.03, 1.15]), and Dadvand et al. [27] (1.17 [1.08, 1.26]), all per 10 µg/m^3^ increase in PM_2.5_ exposure. The two earliest meta-analyses [28,30], as well as three more recent studies [24,33,34], returned non-significant results. Sun et al. [7] also investigated the subgroups within different geographic settings (Table 1), finding the ORs for studies set in the USA to be lower (n = 14 studies, 1.08 [1.02, 1.14]) than the ORs for studies in other countries (n = 5; 1.14 [1.04, 1.25]). Analyses stratified by geographic area in Li et al. [32] resulted in the lowest estimated RR for the Asian subgroup, followed by the Americas and Europe. Socioeconomic status (SES) adjusted estimates were smaller and more precise than unadjusted ORs in Dadvand et al. [27], and even more so when also adjusted for centre-specific covariates, i.e., covariates specific to the locations where the data was generated/collected (e.g., maternal age, ethnicity or smoking). Guo et al. [33] found that ORs for LBW and PM_2.5_ exposure based on central site monitoring network data alone were lower and more precise (1.05 [1.01, 1.09]; n = 7) than those estimated with a land use regression model (1.07 [1.00–1.14]; n = 4). Similar results were found by Sun et al. [7] when using daily monitoring station data (“semi-individual exposure”, non-significant). Results for the “individual exposure” (dispersion models) and “regional exposure assessment” method (calculated by using the regional average PM_2.5_ concentration or a low-resolution grid) were larger, less precise, significant, and based on a smaller number of input studies (data not shown). The overlap between input studies included in the different meta-analyses is limited and no single study was included in all ten meta-analyses.

Four studies investigated the impact of in utero PM_2.5_ exposure on birth weight as a continuous outcome. All four (Table 2) showed significant risks for change in BW (in grams) with increasing PM_2.5_ for non-adjusted or combined analyses (data for PM_10_ show similar results, see Supplementary Materials Table S2). The estimates ranged between −13.9 g [−15.7, −12.06] [29] and −23.4 g [−45.5, −1.4] [30], both per 10 µg/m^3^ PM_2.5_. Of interest is that Sun et al. [7] analysed studies taking place in the USA (n = 13, −18.8 g [−31.4, −6.3]) versus studies from other countries (n = 4, −1.8 g [−12.2, 8.7]) per 10 µg/m^3^ PM_2.5_. The USA subgroup analysis, which showed a greater effect, however, also showed high heterogeneity (99.0%), indicating inconsistencies in the data from the input studies. Stieb et al. [30] demonstrated the biggest change in BW of −23.4 g [−45.5, −1.4] per 10 µg/m^3^ PM_2.5_ for their overall meta-analysis, though with a wide CI. The OR for the studies adjusting for maternal smoking was larger and less precise compared to the estimate for unadjusted studies in Lamichhane et al. [29]. Sun et al. [7] also analysed the exposure assessment subgroups (data not shown) and found results similar to that for the LBW analyses, i.e., the smallest and most precise estimate was for “semi-individual exposure” (significant). “Individual” and “regional exposure assessment” subgroups, which are based on fewer input studies, resulted in larger, less precise and non-significant estimates. The spread of studies included in the meta-analyses was broad with 12 studies included in more than one meta-analysis, of which two were included in all four meta-analyses.

### 3.2. Birth Weight and Later Life CVD Risk

Three meta-analyses were identified from the literature searches (four of the seven studies originally identified were excluded on screening for being out of scope) [36,37,38]. In total there were 47 papers identified across the meta-analyses, although none included more than 70% of these papers and only ten studies were included in all three. Part of the explanation for the discrepancies is the different time periods for which the searches were conducted, and details of the search strategies used, e.g., key word choices and bibliographic databases searched. Despite these inconsistencies, all three meta-analyses showed statistically significant increased risks of CVD with LBW, and two of the analyses identified that the relationship between BW and CVD was non-linear, following a U-shaped form.

Wang et al. [36] focused specifically on coronary heart disease (CHD), including data from 27 papers, and identified that LBW was significantly associated with an increased risk of CHD; a 1 kg increase in BW was associated with an OR of 0.83 [0.80–0.86]. There was significant heterogeneity in these data, although no evidence of publication bias. Knop et al. [37] included 33 studies of CVD involving 5,949,477 participants; a 1 kg increment in BW being associated with an OR of developing CVD of 0.84 [0.81–0.86], with a similar risk of CHD. In this analysis, there was no evidence of heterogeneity. In a categorical analysis of CVD risk by BW, the authors identified a U-shaped pattern, with risk decreasing from the lowest category (<2.5 kg) to 4–4.5 kg, and then increasing in the final category (>4.5 kg). Mohseni et al. [38] also investigated the risk of CVD using data from 24 articles, but with the specific purpose of understanding the change in risk with BW using a restricted cubic spline regression analysis. They identified statistically significant U-shaped non-linearity in the relationship for all cardiovascular disease and for the subtypes coronary heart disease, atrial fibrillation, myocardial infarction and stroke. The OR for CVD for BW >2.5 kg compared to <2.5 kg was 0.88 [0.79–0.97].

## 4. Discussion

There was an increase in risk for LBW with PM_2.5_ exposure during the entire pregnancy (Table 1), with ORs/RRs per 10 µg/m^3^ increase in PM_2.5_ ranging from 1.04 [0.99, 1.09] to 1.17 [1.08, 1.26], with the exception of Guo et al. [33] who found no effect. However, a clear trend was observed for the BW with PM_2.5_ exposure. All meta-analyses (Table 2), except the two subgroup analyses with the smallest number of input studies, showed significant decreases in BW per 10 µg/m^3^ increase in PM_2.5_ ranging from −1.8 g [−12.2, 8.7] to −23.4 g [−45.5, −1.4].

The methodological quality of the contributing studies is a potential source of confounding. Subgroup analyses have shown that better quality studies yield statistically significant and larger effect estimates and/or less heterogeneity, compared to studies rated as lower quality [29,33]. Several authors noted that the degree of heterogeneity in the associations varied considerably by pollutant, outcome and exposure period, although there were often insufficient studies available to investigate sources of heterogeneity when looking at subgroups such as the study region or exposure assessment method [24,30]. In general, analyses based on higher numbers of input studies resulted in more precise estimates. The contributing studies usually rely on birth records, which hold limited individual-level information, to control for potential confounding factors such as SES or access to prenatal care [28,39]. Adjusting or not adjusting for SES is a source of heterogeneity amongst the input studies identified by Zhu et al. [31]. Maternal smoking is a known risk factor for adverse pregnancy outcomes and thus an important potential confounder [4], but most studies were not able to adjust for this because maternal smoking status was mostly not recorded in the original research. We accept that BW is not necessarily the best measure to use as a link between in-utero exposure to particulate air pollution and later life health outcomes, and preterm birth or intrauterine growth restriction might be better. However, we judged that the data in the published literature was not sufficient to sustain the use of such measures.

The PM and BW meta-analyses considered here generally relied on fixed site air pollution measurements near the residence of the mother, while fewer studies used modelled PM data, such as data from, e.g., dispersion or land use regression models, or included PM exposure assessments from satellite data [24,31,32]. Such data are surrogates for personal exposure and considerable differences in temporal resolutions and the distance of maternal home address to the monitoring sites exist between individual studies, which may lead to exposure misclassification [40]. Maternal mobility during pregnancy and the lack of indoor air quality information further adds to potential misclassification [24,28]. Subgroup analyses investigating differences between estimates based on various exposure assessment methods indicate that those based on ground-level monitoring data (for which most literature are available) are more precise. Thus, any results need to be interpreted with the potential for exposure misclassification in mind, and future studies should explore improved, more person-centred, exposure assessment approaches.

However, despite the limitations in the available data, we concluded that in utero exposure to outdoor PM air pollution is associated with a small reduction in BW. For the purposes of this review and based on our previous evaluation of the evidence for the toxicity of PM from biomass burning [41], we assumed that PM is equally toxic to the foetus regardless of the source or setting.

There is persuasive evidence for an association between BW and CVD in later life, with the least risk in infants weighing 4 to 4.5 kg and greater risks for lower and higher BWs. For LBW children, a 1 kg decrease in BW is associated with an OR for CVD of around 0.84. The possible biological mechanisms to explain these associations are unclear but could include the effect of exposure of the foetus to environmental pollutants (e.g., PM), genetic factors, or a combination of these or other environmental, behavioural or nutritional factors. However, the overall evidence for foetal programming rather than LBW causing the subsequent risk of non-communicable disease is less certain. We chose to assume that regardless of the particular cause or mechanism causing LBW, the resulting health impacts are likely to be the same. The changes in BW linked to PM pollution will likely have a small impact on CVD risk, but potentially affect a large number of people, which makes these exposures important from a public health perspective, particularly in terms of the impact on morbidity in later life. For example, the population risk of cardiovascular mortality is estimated to be 0.6% higher from 40 µg/m^3^ PM_2.5_ in utero exposure compared to 30 µg/m^3^, based on an average 24 g decrease in BW [30] and the risk estimate from Wang et al. [36]. This compares to an 11% increased relative risk (1.62 to 1.80) for cardiovascular mortality from adult exposure to the same level of PM_2.5_ through the life course, based on the Global Estimates of Mortality Model (GEMM) [42].

## 5. Conclusions

We have reviewed meta-analyses, which analyse the effects of PM exposure in utero on birth weight and subsequently, the effects of lower BW on CVDs in old age. In the first part we found the data support a small reduction of BW related to PM exposure in utero over the entire pregnancy. In the second part we found evidence for an association between BW and later life CVD, but biological mechanisms for this association remain unclear. Overall, the effect of PM pollution in utero on BW are so small that the effects on later life CVD will likely also be very small, although they potentially affect a large number of people. The mechanistic evidence to support the impact of in utero PM exposure on BW and subsequent later life cardiovascular health is weak and further research is needed. Additional epidemiologic studies of BW in relation to PM exposure, with improved exposure assessment and better assessment of confounding factors, should be undertaken. In addition, studies are needed to investigate the risk of CVD from exposure to PM across the whole life course.

## Figures and Tables

**Table 1:** Meta-analyses results for LBW or term low birth weight (TLBW) (*) and PM_2.5_ exposure during the entire pregnancy.

Descriptive Information on Meta-Study and All Input Studies				Meta-Risk Estimates for Specific Analyses			
Reference	Country/Region	Range of Participants/Births	Exposure Range (Using Study Metrics)	Adjusted for/Subgroup	Studies Included in Risk Estimate Analysis (n)	OR/RR (95% CI), PM_2.5_ per 10 µg/m^3^ Increment	Heterogeneity (I^2^)
Sapkota et al. 2012 (*) [28]	USA (n = 9), Canada and Taiwan (n = 2 each), Germany, Brazil, Korea and (n = 1 each)	Participants: 128 to 374,167; Cases: 69 to 21,450	Range of PM_2.5_ means (where reported): 5.1–11.9 µg/m^3^; Range of PM_2.5_ medians (where reported): 13.5–14.4 µg/m^3^	NA	4	1.09 [0.90, 1.32]	57.4%
Stieb et al. 2012 [30]	North America (n = 27), Europe (n = 18), Asia (n = 10), Australia (n = 4), South America (n = 3)	Births: 153 to 3,303,834	PM_2.5_ Min./Max. average 24 h concentrations (µg/m^3^) among all studies: Min. 1.8 Max. 44.2	NA	6	1.05 [0.99, 1.12]	85.5%
Dadvand et al. 2013 (*) [27]	North America (6), Europe (5), South America (1), Asia (1), Oceania (1)	Births: ~1000 to ~2 m	Range of PM_2.5_ medians (where reported): 3.98–20.3 µg/m^3^	NA	7	1.17 [1.08, 1.26]	92.3%
				Adjusted for maternal SES		1.10 [1.03, 1.18]	89.7%
				Adjusted for maternal SES and centre specific covariates **		1.04 [0.99, 1.09]	68.5%
Zhu et al. 2015 [31]	USA (n = 17), Canada (n = 2), Australia (n = 2), Netherlands, UK, Poland and Norway (n = 1 each)	Study population: 481 to 1,5480,904	NA	NA	6	1.05 [1.02, 1.07]	39.70%
Sun et al. 2016 [7]	USA (n = 23), Multi-country (n = 2), Canada, Spain, Netherlands, UK, Poland, Norway, Australia (n = 1 each)	Participants: 481 to 3,545,177	Range of PM_2.5_ (where reported): 5.1–43.8 µg/m^3^; Except (Fleischer et al., 2014 [35]): 1.4–98.1 µg/m^3^		19	1.09 [1.03, 1.15]	92.6%
				USA	14	1.08 [1.02, 1.14]	94.3%
				Other setting	5	1.14 [1.04, 1.25]	36.1%
Li et al. 2017 (*) [24]	USA n = 17, Canada n = 2, Europe n = 3, other n =1	Births: 3853 to 3,545,177	Range of PM_2.5_ means (where reported) 1.82–22.11 µg/m^3^	NA	4	1.05 [0.98, 1.12]	85.0%
Guo et al. 2019 [33]	USA (n = 17), Canada (n = 4), Brazil and China (n = 3 each), Spain, Australia, South Korea, and multi-country (n = 2 each), Iran, UK, Taiwan, Norway, Sweden, and Japan (n = 1 each)	Births: 225 to 2,402,545	NA	NA	6	1.00 [0.98, 1.03]	73.3%
Ji et al. 2019 [34]	USA and Canada (n = 3 each), Iran, Korea, Netherlands, Taiwan, Brazil, UK, Spain, multi-country (n = 1 each)	Births: 225 to 423,719	NA	NA	6	1.04 [0.99, 1.09]	67.4%
Li et al. 2020 *** [32]	USA (n = 20), China (n = 9), Korea (n = 5), Canada (n = 4), Japan, Lithuania, Spain, UK (n = 2 each), Australia, Czech republic, India, Iran, Peru, Poland, Puerto Rico, multi-country (n = 1 each)	225 to 3,545,177	NA		29	1.08 [1.04, 1.12]	86.0%
				The Americas	18	1.07 [1.02, 1.12]	>50%
				Asia	7	1.04 [0.99,1.10]	>50%
				Europe	4	1.38 [1.19, 1.59]	<50%

* term low birth weight. ** centre-specific covariates = covariates specific to the locations where the data were generated/collected. *** the authors report relative risk (RR) estimates.

**Table 2:** Meta-analyses results for change in BW (g) and PM_2.5_ exposure during the entire pregnancy.

Descriptive Information on Meta-Study and All Input Studies				Meta-Risk Estimates for Specific Analyses			
Reference	Country/Region	Range of Participants/Births	Exposure Range (using Study Metrics)	Adjusted for/Subgroup	Studies Included in Risk Estimate Analysis (n)	Change in BW (g) (95% CI), PM_2.5_ per 10 µg/m^3^ Increment	Heterogeneity (I^2^)
Stieb et al. 2012 [30]	North America (n = 27), Europe (n = 18), Asia (n = 10), Australia (n = 4), South America (n = 3)	Births: 153 to 3,303,834	PM_2.5_ Min./Max. average 24-hour concentrations (µg/m^3^) among all studies: Min. 1.8 Max. 44.2		7	−23.4 [−45.5, −1.4]	94.7%
Lamichhane et al. 2015 [29]	North America (n = 25), Asia (n = 7), Europe (n = 6), Australia (n = 4), and South America (n = 2)	Births: 235 to 3,303,834	Range of PM_2.5_ means (where reported): 9.4–21.3 µg/m^3^	Combined studies *	8	−13.88 [−15.7, −12.06]	47.5%
				Adjusted for maternal smoking	7	−22.17 [−37.93, −6.41]	92.3%
Zhu et al. 2015 [31]	USA (n = 17), Canada (n = 2), Australia (n = 2), Netherlands, UK, Poland and Norway (n = 1 each)	Population: 481 to 1,548,904	NA		12	−14.58 [−19.31, −9.86]	86.8%
Sun et al. 2016 [7]	USA (n = 23), Multi-country (n = 2), Canada, Spain, Netherlands, UK, Poland, Norway, Australia (n = 1 each)	Participants: 481 to 3,545,177	Range of PM_2.5_ means (where reported): 5.1–43.8 µg/m^3^; Except (Fleischer et al., 2014 [35]): 1.4–98.1 µg/m^3^		17	−15.9 [−26.8, −5.0]	98.5%
				USA	13	−18.8 [−31.4, −6.3]	99.0%
				Other settings	4	−1.8 [−12.2, 8.7]	26.2%

* combined maternal smoking adjusted and non-adjusted studies due to the lack of non-adjusted studies.

## Supplementary Materials

The following are available online at https://www.mdpi.com/1660-4601/17/23/8948/s1, Table S1: Meta-analyses results for LBW or TLBW and PM_10_ exposure during the entire pregnancy, Table S2. Meta-analyses results for change in BW (g) and PM_10_ exposure during the entire pregnancy.
