# Supplementary material for: In Utero Exposure to Particulate Air Pollution during Pregnancy: Impact on Birth Weight and Health through the Life Course
Source: Int J Environ Res Public Health. 2020 Dec 1;17(23):8948. doi: 10.3390/ijerph17238948 (PMC7730886; doi:10.3390/ijerph17238948)
Supplement: Supplementary file 1 [file ijerph-17-08948-s001.pdf]

International Journal of
Environmental Research and Public Health

MDPI

Table S1. Meta-analyses results for LBW or TLBW (*) and PM_{10} exposure during the entire pregnancy.

Descriptive information on meta-study and all input studies				Meta-risk estimates for specific analyses			
**Reference**	**Country/Region**	**Range of participants/ births**	**Exposure range (using study metrics)**	**Adjusted for/subgroup**	**Studies included in risk estimate analysis (n)**	**OR/RR [95% CI], PM_{10} per 10 μg/m³ increment**	**Heterogeneity (I^2)**
Sapkota et al. 2012(*) [28]	USA (n = 9), Canada and Taiwan (n = 2 each), Germany, Brazil, Korea and (n = 1 each)	Population: 128 to 374,167; Cases: 69 to 21,450	Range of PM_{10} means (where reported): 12.5-71.1 μg/m³; Range of PM_{10} medians (where reported): 7.84-30.1 μg/m³	NA	11	1.02 [0.99, 1.06]	54.5%
Stieb et al. 2012 [30]	North America (n=27), Europe (n=18), Asia (n=10), Australia (n=4), South America (n=3)	Births: 153 to 3,303,834	PM_{10} Min. /Max. average 24-hour concentrations (μg/m³) among all studies: Min. 3.3 Max. 89.7	NA	14	1.05 [1.02, 1.07]	68.4%
Dadvand et al. 2013(*) [27]	North America (6), Europe (5), South America (1), Asia (1), Oceania (1)	Births: ~1,000 to ~2 m	Range of PM_{10} medians(where reported): 12.5-66.5 μg/m³	NA	13	1.04 [1.01, 1.06]	76.5%
				adjusted for maternal SES		1.03 [1.01, 1.05]	79.4%
				adjusted for maternal SES and centre specific covariates**		1.02 [1.01, 1.04]	54.3%
Guo et al. 2019 [31]	USA (n = 17), Canada (n = 4), Brazil and China (n = 3 each), Spain, Australia, South Korea, and multi-country (n = 2 each), Iran, UK, Taiwan, Norway, Sweden, and Japan (n = 1 each)	Births: 225 to 2,402,545	NA		11	1.03 [1.01, 1.04]	73.3%
				adjusted for maternal smoking	6	1.03 [1.01, 1.05]	68.6%
				not adjusted for maternal smoking	5	1.01 [0.98, 1.05]	78.0%
				NOS*** score ≤ 7	3	0.99 [0.93, 1.04]	76.8%
				NOS*** score ≥ 7	8	1.07 [1.03, 1.11]	62.3%
				Asian setting	2	0.93 [0.84, 1.02]	30.2%
				Non-Asian setting	9	1.03 [1.01, 1.05]	74.0%

Ji et al. 2019 [34]	**USA and Canada (n = 3 each), Iran, Korea, Netherlands, Taiwan, Brazil, UK, Spain, multi-country (n = 1 each)**	**Births: 225 to 423,719**	**NA**		**9**	**1.01 [0.96, 1.08]**	**67.5%**
				Asia	3****	0.98 [0.90, 1.07]	48.6%
				Europe and America	9	1.05 [1.01, 1.09]	54.2%
Li et al. 2020 *****[32]	USA (n= 20), China (n = 9), Korea (n = 5), Canada (n = 4), Japan, Lithuania, Spain, UK (n = 2 each), Australia, Czech republic, India, Iran, Peru, Poland, Puerto Rico, multi-country (n = 1 each)	225 to 3,545,177	NA		23	1.05 [1.03, 1.08]	70.3%
				The Americas	6	1.02 [0.97, 1.07]	>50%
				Asia	14	1.05 [1.02, 1.08]	>50%
				Europe	3	1.11 [1.07, 1.17]	<50%

** Centre specific covariates = covariates specific to the locations where the data was generated/collected

*** Newcastle-Ottawa quality score for assessing the quality of nonrandomised studies in meta-analyses (http://www.ohri.ca/programs/clinical_epidemiology/oxford.asp)

**** We assume the following studies have been analysed as "Asia" subgroup: [1-3]

***** The authors report Relative Risk (RR) estimates

International Journal of Environmental Research and Public Health

MDPI

Table S2. Meta-analyses results for change in BW (g) and PM_{10} exposure during the entire pregnancy.

Descriptive information on meta-study and all input studies				Meta-risk estimates for specific analyses			
**Reference**	**Country/Region**	**Range of births/participants**	**Exposure range (using study metrics)**	**Adjusted for/subgroup**	**Studies included in risk estimate analysis (n)**	**change in BW [g] [95% CI] PM_{10} per 10 μg/m³ increment**	**Heterogeneity (I^2)**
Stieb et al. 2012 [30]	North America (n=27), Europe (n=18), Asia (n=10), Australia (n=4), South America (n=3)	Births: 153 to 3,303,834	PM_{10} Min. /Max. average 24-hour concentrations (μg/m³) among all studies: Min. 3.3 Max. 89.7		7	-8.4 [-10.1, -6.65]	15.9%
Dadvand et al. 2013 [27]	North America (n=6), Europe (n=5), South America (n=1), Asia (n=1), Oceania (n=1)	Births: ~1,000 to ~2 m	Range of PM_{10} (where reported): 12.5-66.5 μg/m³		11	-2.7 [-7.2, 1.7]	NA
				adjusted for maternal SES	11	-3.0 [-6.9, 0.9]	NA
				adjusted for maternal SES and centre specific covariates*	11	-8.9 [-13.2, -4.6]	NA
Lamichhane et al. 2015 [29]	North America (n=25) , Asia (n=7), Europe (n=6), Australia (n=4), and South America (n=2)	Births: 235 to 3,303,834	Range of PM_{10} means (where reported): 2.97-89.7 μg/m³	combined studies	8	-6.50 [-10.94, -2.5]	76.4%
				adjusted for maternal smoking	5	-10.31 [-13.57, -7.05]	0.0%
				not adjusted for maternal smoking	3	-8.17 [-10.99, -5.36]	35.2%
				relatively better quality studies**	5	-10.59 [-13.24, -7.94]	0.0%
				relatively low quality studies**	4	-2.86 [-12.35, 6.64]	89.9%

* Centre specific covariates = covariates specific to the locations where the data was generated/collected

** Quality assessment based on checklist by [4]

International Journal of Environmental Research and Public Health

MDPI

1. Araban, M.; Kariman, N.; Tavafian, S.; Motesaddi, S.; Alavimajd, H.; Shokravi, F.A. Air Pollution and Low Birth Weight: A Historical Cohort Study from Tehran/Pollution Atmospherique Et Faible Poids De Naissance: Une Etude De Cohorte Historique a Teheran. Eastern Mediterranean Health Journal **2012**, *18*, 556-561.

2. Kim, O.; Ha, E.; Kim, B.; Seo, J.; Park, H.; Jung, W.; Lee, B.; Suh, Y.; Kim, Y.; Lee, J. PM10 and Pregnancy Outcomes: A Hospital-Based Cohort Study of Pregnant Women in Seoul. Journal of occupational and environmental medicine **2007**, *49*, 1394-1402.

3. Lin, C.; Li, C.; Yang, G.; Mao, I. Association between Maternal Exposure to Elevated Ambient Sulfur Dioxide during Pregnancy and Term Low Birth Weight. Environ. Res. **2004**, *96*, 41-50.

4. Downs, S.H.; Black, N. The Feasibility of Creating a Checklist for the Assessment of the Methodological Quality both of Randomised and Non-Randomised Studies of Health Care Interventions. J. Epidemiol. Community Health **1998**, *52*, 377-384.
